# 3D Spatial Combination of CN Vacancy‐Mediated NiFe‐PBA with N‐Doped Carbon Nanofibers Network Toward Free‐Standing Bifunctional Electrode for Zn–Air Batteries

**DOI:** 10.1002/advs.202105925

**Published:** 2022-02-22

**Authors:** Chenglong Lai, Haomiao Li, Yi Sheng, Min Zhou, Wei Wang, Mingxing Gong, Kangli Wang, Kai Jiang

**Affiliations:** ^1^ State Key Laboratory of Advanced Electromagnetic Engineering and Technology School of Electrical and Electronic Engineering Huazhong University of Science and Technology Wuhan 430074 P. R. China; ^2^ School of Materials Science and Engineering Huazhong University of Science and Technology Wuhan Hubei 430074 China; ^3^ Engineering Research Center of Power Safety and Efficiency Ministry of Education Wuhan Hubei 430074 China; ^4^ Engineering Research Center of Nano‐Geomaterials of Ministry of Education Faculty of Materials Science and Chemistry China University of Geosciences Wuhan 430078 China

**Keywords:** CN vacancy, flexible 3D free‐standing, flexible Zn–air battery, N_2_‐plasma

## Abstract

Constructing flexible free‐standing electrodes with efficient bifunctional performance is significant for improving the performance of flexible Zinc–air batteries. Herein, a flexible free‐standing bifunctional electrode (N_2_‐NiFe‐PBA/NCF/CC‐60) is constructed by the 3D spatial combination of CN vacancy‐mediated NiFe Prussian Blue Analogue (NiFe‐PBA) and N‐doped carbon nanofibers (NCF) rooted on carbon cloth (CC). The in situ formed CN vacancies by N_2_‐plasma activation tune the local coordination environment and electronic structure of Ni‐Fe active sites in NiFe‐PBA, thus improving the oxygen evolution reaction (OER) catalytic intrinsic activity, and restraining the loss of Fe element during OER process. The combination of NiFe‐PBA and NCF presents a 3D interworking network structure, which exhibits a large specific surface and excellent electrical conductivity, thus guaranteeing sufficient, stable, and efficient oxygen reduction reaction (ORR)/OER active sites. Therefore, the N_2_‐NiFe‐PBA/NCF/CC‐60 electrode delivers high‐efficiency OER activity with a low overpotential (270 mV at 50 mA cm^−2^) and excellent ORR performance with a positive potential of 0.89 V at 5 mA cm^−2^. The N_2_‐NiFe‐PBA/NCF/CC‐60 based Zn–air batteries display outstanding discharge/charge stability for 2000 cycles. Meanwhile, the corresponding flexible Zn–air batteries with satisfactory mechanical properties exhibit a low voltage gap of 0.52 V at 1.0 mA cm^−2^.

## Graphical abstract

A flexible free‐standing bifunctional electrode (N_2_‐NiFe‐PBA/NCF/CC‐60) is constructed by the 3D spatial combination of CN vacancy‐mediated NiFe‐PBA and N‐doped carbon nanofibers rooted on carbon cloth. The N_2_‐NiFe‐PBA/NCF/CC‐60 electrode delivers high‐efficiency and stabilized bifunctional oxygen reduction and oxygen evolution reaction catalytic performance. Furthermore, the N_2_‐NiFe‐PBA/NCF/CC‐60 based Zn–air battery operates 2000 discharge/charge cycles.

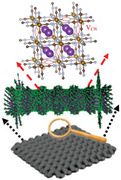

## 1 Introduction

Flexible electronics have been gradually applied to many industries ranging from consumer products, medical and automotive to aerospace, due to their prominent advantages of flexibility, foldability, and potential wearability. Therefore, developing well‐matched flexible energy storage/conversion devices is crucial for fulfilling a “flexible electronics” society. Rechargeable Zinc–air battery has been deemed as one of the most promising energy storage devices for portable electronics, especially for flexible electronics, due to good security and high theoretical specific energy density.^[^ ^1^, ^2^, ^3^ ^]^ However, the oxygen evolution and oxygen reduction reaction (OER/ORR) in air‐electrode during charge/discharge of Zinc–air battery are accompanied by multiple proton‐coupled electron transfer. The ORR/OER always suffers from sluggish reaction kinetics, thus causing the huge charge/discharge overpotential and poor battery performance. Exploring the catalysts with excellent OER/ORR catalytic activity and durability is one of the most effective strategies to promote Zn–air battery performance. ^[^ ^4^, ^5^, ^6^ ^]^ On the other hand, the integration of catalysts and air electrodes will inevitably lead to the introduction of the binder. The binder with poor electrical conductivity will cover the surface of the material, which lowers the surface reactive sites and increases the resistance of the electrode, thus weakening the electrocatalysis performance of catalysts. Besides, the possible deterioration of the binder would affect the long‐term operation of the air‐electrode. In response, constructing a binder‐free electrode based on high‐efficiency OER/ORR catalysts and a 3D skeleton can simultaneously solve these problems.

Prussian blue analogs (PBAs) are a class of perovskite‐type materials with a unique structure, and its molecular formula is generally described as A*_x_* M[Fe(CN)_6_]*_y_* ^.^ mH_2_O (*y* < 1, 0 < *x* <2), where M is transition metal and A is alkali metal.^[^ ^7^, ^8^, ^9^, ^10^, ^11^, ^12^ ^]^ Currently, the PBAs have emerged as catalysts or procatalysts for OER. Many researchers have studied the microstructure and/or catalytic mechanism of PBAs to further improve the catalytic activity.^[^ ^13^, ^14^, ^15^, ^16^, ^17^ ^]^ For example, Galan‐Mascaros et al.^[^ ^18^ ^]^ verified that Co‐Fe PBA catalyst exhibits more efficient and stable catalytic performance for OER after chemical etching treatment. Thereinto, manipulating the defect chemistry is a popular and efficient strategy to tune the catalytic activity of materials. The vacancy is the most common and easily regulated defect, which can not only tailor the charge concentration and surface electronegativity but also preserve the crystal structure of materials, thus promoting catalytic activity and stability. For instance, Fan et al.^[^ ^19^ ^]^ synthesized double‐shelled NiFe‐PBA with CN vacancies (V_CN_) through thermally activated strategy, which exhibits more efficient OER catalytic activity. However, it is still necessary to explore simple and effective strategies to form vacancies on PBAs to enhance catalytic performance.

Besides, the PBAs are difficult to be used as bifunctional catalytic materials, due to the poor ORR activity. Constructing composite catalysts is one of the most efficient strategies to prepare PBAs‐based bifunctional catalysts. The heteroatoms doped carbon materials with unique microstructure can be used as a carrier for the growth of PBAs and display efficient ORR activity, which is one of the best composite materials.^[^ ^20^, ^21^, ^22^ ^]^

However, there is still a big challenge to integrate PBAs and heteroatoms doped carbon as an air‐electrode for flexible Zinc–air batteries, meantime, further improving the catalytic performance by manipulating defect chemistry.^[^ ^23^, ^24^, ^25^, ^26^ ^]^

Here, a flexible binder‐free air‐electrode consisting of V_CN_‐mediated NiFe‐PBA, N‐doped carbon nanofibers (NCF) and carbon cloth (CC) is synthesized by N_2_‐plasma activation (60 min) of NiFe‐PBA which grew on NCF/CC. The existence of V_CN_ in NiFe‐PBA is confirmed by the high‐resolution transmission electron microscope (HRTEM), Raman, and electron spin resonance. The V_CN_ greatly improves the OER catalytic intrinsic activity of N_2_‐NiFe‐PBA/NCF/CC‐60 and restrains the loss of Fe element during the OER process. A large number of V_CN_‐mediated NiFe‐PBA grow on NCF/CC with a 3D network structure, providing adequate, stable, and high‐efficiency ORR/OER catalytic active sites. Therefore, the N_2_‐NiFe‐PBA/NCF/CC‐60 electrode delivers high‐efficiency and stabilized bifunctional (OER/ORR) performance. Furthermore, the N_2_‐NiFe‐PBA/NCF/CC‐60 based liquid Zn–air battery operates 2000 discharge/charge cycles. The flexible Zn–air batteries manifest satisfactory battery performance and mechanical properties.

## 2 Results and Discussion

### 2.1 Morphological and Structural Characterization

**Scheme 1:**
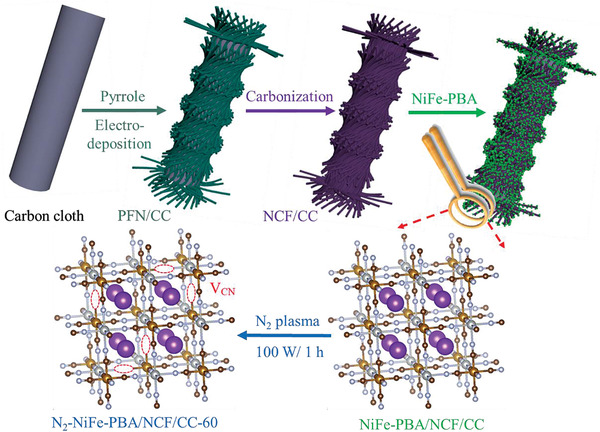
Schematic diagram of the synthesis of N_2_‐NiFe‐PBA/NCF/CC‐60.

**Figure 1:**
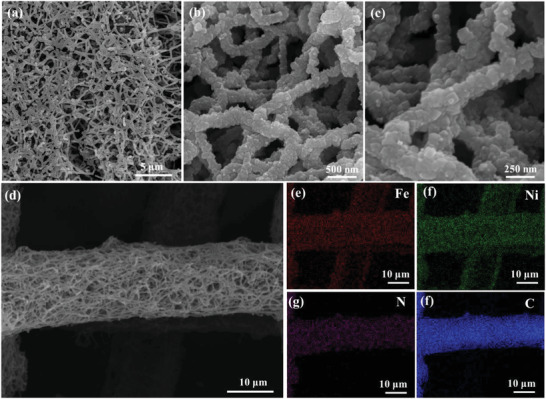
a–d) The SEM images of N_2_‐NiFe‐PBA/NCF/CC‐60. EDS elements mapping images of Fe e), Ni f), N g), and C f) for N_2_‐NiFe‐PBA/NCF/CC‐60.

The schematic diagram of the synthesis of N_2_‐NiFe‐PBA/NCF/CC‐60 electrode is figuratively shown in **Scheme** **1**. First, the 3D PPy nanofibers rooted on CC were prepared by constant voltage electrodeposition. Second, the N‐doped carbon fibers on CC were synthesized via a high‐temperature carbonization process (NCF/CC). Third, the NiFe‐PBA was uniformly grown on the substrate of NCF/CC (NiFe‐PBA/NCF/CC). Finally, the N_2_‐NiFe‐PBA/NCF/CC‐60 electrode with V_CN_ is successfully constructed via N_2_‐plasma activation of the NiFe‐PBA/NCF/CC for 60 min. For comparison, the N_2_‐NiFe‐PBA/NCF/CC‐X with different activation times of N_2_‐plasma (30, 60, and 120 min) was prepared under the same procedure. The morphologies and structures of the N_2_‐NiFe‐PBA/NCF/CC‐60 and precursors are performed by scanning electron microscopy (SEM). It is seen from **Figure** **1**a–c that plenty of regular nanocubes wrap the N‐doped carbon nanofibers, exhibiting a unique 3D network structure of the N_2_‐NiFe‐PBA/NCF/CC‐60. The distribution of elements of the electrode is characterized by SEM‐energy dispersive spectrometer (SEM‐EDS) element mapping. As shown in (Figure 1d–f), Fe, Ni, and N elements are uniformly dispersed in carbon cloth, which indicates the homogeneous 3D spatial combination of NiFe‐PBA with NCF in N_2_‐NiFe‐PBA/NCF/CC‐60. Furthermore, the unique 3D network structure is also clearly observed in NCF/CC (Figure S1, Supporting Information). The microstructure of the NiFe‐PBA/NCF/CC and NiFe‐PBA/CC are comparatively analyzed via SEM to reveal the effects of the NCF. Compared with NiFe‐PBA/NCF/CC, the scattered NiFe‐PBA nanocubes are attached to CC in NiFe‐PBA/CC (Figures S2 and S3, Supporting Information). These results indicate NCF with 3D network structure and large specific surface benefit to the growth of NiFe‐PBA.^[^ ^27^, ^28^ ^]^ Besides, after plasma treatment for 30/60 min, the hexahedral microstructure of NiFe‐PBA and the 3D network structure of NCF are still observed and maintained (Figure 1a–c; and Figure S4, Supporting Information). However, the carbon fibers get rupture and the morphology of NiFe‐PBA becomes irregular in the surface of N_2_‐NiFe‐PBA/NCF/CC‐120 (Figure S5, Supporting Information). Moreover, the X‐ray diffraction (XRD) patterns of the obtained samples are shown in Figure S6 (Supporting Information), the NiFe‐PBA/NCF/CC and N_2_‐NiFe‐PBA/NCF/CC‐X exhibit the pure cubic K_2_NiFe(CN)_6_ phase (JCPDS no. 20‐0915), indicating that the crystal structure is not affected by N_2_‐plasma activation. These results verify that N_2_‐plasma activation does not affect the phase and component of NiFe‐PBA/NCF/CC, but the microtopography in the surface of NiFe‐PBA/NCF/CC will be destroyed after overlong N_2_‐plasma activation.

**Figure 2:**
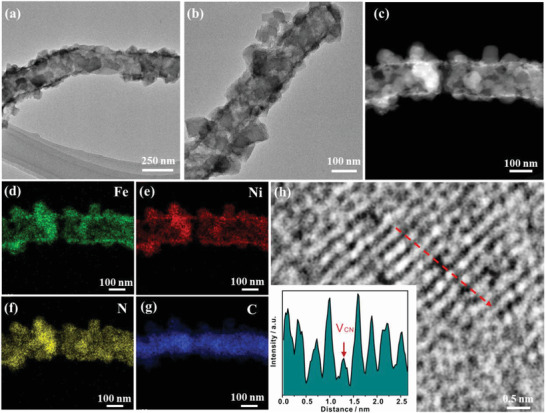
a–b) TEM images of N_2_‐NiFe‐PBA/NCF/CC‐60. STEM image c) and elemental mapping images of Fe d), Ni e), N f), and C g). h) HRTEM image of N_2_‐NiFe‐PBA/NCF/CC‐60, Inset is the atomic intensity profile along the red line in h).

For further investigating the N_2_‐NiFe‐PBA/NCF/CC‐60, the microstructure and morphology of the sample were studied by TEM. **Figure** **2**a,b shows that NiFe‐PBAs with hexahedral microstructure are evenly adsorbed on the carbon fibers. As shown in Figure 2c–g, Ni/Fe elements and C element are mainly concentrated on the outside and inside of materials, respectively. The N element is evenly distributed throughout the material. This phenomenon is consistent with the microstructure of N_2_‐NiFe‐PBA/NCF/CC‐60. Besides, the crystalline domains with the discontinuous atomic arrangement are observed in the HRTEM image of N_2_‐NiFe‐PBA/NCF/CC‐60 (Figure 2h), which is also confirmed by the peak valleys of the atomic intensity profile (inset in Figure 2h). These results reveal the presence of defects which could be V_CN_.

**Figure 3:**
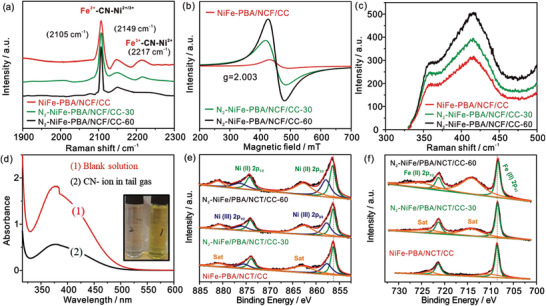
a) Raman, b) ESR spectra, and c) PL spectra of NiFe‐PBA/NCF/CC, N_2_‐NiFe‐PBA/NCF/CC‐X. d) The UV signals of the tail gas absorbed solution. The fine Ni spectra e) and Fe spectra f) of NiFe‐PBA/NCF/CC and N_2_‐NiFe‐PBA/NCF/CC‐X.

Raman spectroscopy is conducted to demonstrate the presence of V_CN_. The two peaks at 2105 and 2149 cm^−1^ are clearly shown in all Raman curves (**Figure** **3**a), which correspond to the vibrations of CN groups in the mixture of Fe^2+^‐CN‐Ni^3+^ and Fe^2+^‐CN‐Ni^2+^.^[^ ^29^, ^30^ ^]^ The peak at 2217 cm^−1^ attributed to the CN vibrations in Fe^3+^‐CN‐Ni^2+^ tends to disappear gradually with the increase of N_2_‐plasma activation time, indicating the change of state of Fe^3+^‐CN‐Ni^2+^ units. Therefore, the V_CN_ could be attributed to the cleavage of Ni‐N/Fe‐C bonds in Fe^3+^‐CN‐Ni^2+^ units under the N_2_‐plasma activation process. Furthermore, the electron spin resonance (ESR) signal with a g‐value of 2.003 is derived from the unpaired electrons attributed to vacancies, which tends to be more obvious with the increase of time (Figure 3b).^[^ ^31^ ^]^ This result shows that V_CN_ gradually increases under the bombardment of N_2_‐plasma. Considering that the photoluminescence (PL) signal is affected by the vacancies,^[^ ^32^ ^]^ we conducted PL measurement on N_2_‐NiFe‐PBA/NCF/CC‐60. An increasing tendency of PL signals can be observed in Figure 3c, attributing to the high quantum efficiency of excitons localized at the V_CN_ sites. Finally, the tail gas during N_2_‐plasma activation is collected and injected into the ninhydrin and Na_2_CO_3_ mixed solution. The observed fading phenomenon and the reduced ultraviolet (UV) absorption signal confirm the escape of the CN units from NiFe PBAs (Figure 3d).^[^ ^33^ ^]^ In conclusion, all of the above results indicate the formation of V_CN_ in N_2_‐NiFe‐PBA/NCF/CC‐60.

The change of valence state of as‐prepared materials before and after N_2_‐plasma activation is confirmed by X‐ray photoelectron spectroscopy (XPS) measurement. As shown in Figure 3e, the high‐resolution spectra of Ni of NiFe‐PBA/NCF/CC and N_2_‐NiFe‐PBA/NCF/CC‐X are fitted into three components corresponding to Ni (II) 2p, Ni (III) 2p, and sat peaks, respectively. It is observed from Figure 3e; and Figure S7a (Supporting Information) that the binding energy of Ni 2p peaks increases gradually with the increase of N_2_‐plasma activation time. Meantime, it can be concluded from Figure 3f; and Figure S7b (Supporting Information) that Fe peaks shift to the lower binding energy. The increase of Ni valence state and the decrease of Fe valence state indicate the partial electron transfer between Fe and Ni, thus demonstrating that the in situ formed CN vacancies tune the local coordination environment and electronic structure of the Ni‐Fe active sites. Figure S8a (Supporting Information) shows the fine N spectra of NCT/CC, which exhibits three characteristic peaks corresponding to N—O, graphitic‐N, and pyridinic‐N, respectively. As shown in Figure S8b–d (Supporting Information), the fine N spectra mainly manifest as pyrrolic‐N, which is assigned to the N structure in NiFe‐PBA. Furthermore, the Brunauer‐Emmett‐Teller (BET) measurement is conducted to acquire the specific surface area of the N_2_‐NiFe‐PBA/NCF/CC‐60 electrode. As shown in Figure S9 (Supporting Information), the N_2_‐NiFe‐PBA/NCF/CC‐60 electrode exhibits a special surface area of 27.8 m^2^ g^−1^, higher than that of N_2_‐NiFe‐PBA/CC‐60 (6.6 m^2^ g^−1^), demonstrating the large specific surface area.

### 2.2 Electrocatalytic Performance of N_2_‐NiFe‐PBA/NCF/CC‐60 Electrode

**Figure 4:**
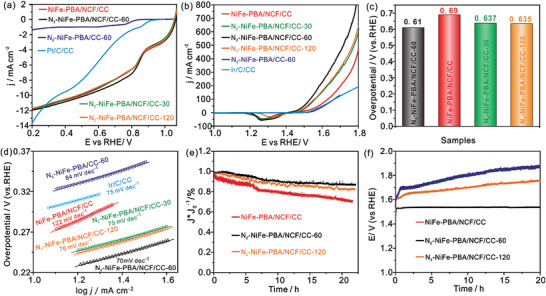
The ORR a) and OER b) polarization curves of catalysts. c) The histogram of overall overpotential. d) The OER Tafel curves of catalysts. e) 22 h chronoamperometric measurement of catalysts at 0.70 V. f) The voltage‐time curves of catalysts at 20 mA cm^−2^.

The electrocatalytic performance of N_2_‐NiFe‐PBA/NCF/CC‐60 is investigated by the electrochemical workstation. As shown in **Figure** **4**a, compared with N_2_‐NiFe‐PBA/CC‐60 and Pt/C/CC, the N_2_‐NiFe‐PBA/NCF/CC‐X and NiFe‐PBA/NCF/CC exhibit much more high‐efficiency ORR catalytic activity with a positive potential of 0.89 V at 5.0 mA cm^−2^, attributed to the fact that NCT with 3D connected network structure not only is beneficial for air transfer but also guarantees sufficient and efficient ORR active sites. Furthermore, N_2_‐NiFe‐PBA/NCF/CC‐X exhibit more prominent OER activity relative to NiFe‐PBA/NCF/CC and Ir/C/CC, demonstrating the formation of V_CN_ in NiFe‐PBA by N_2_‐plasma activation is responsible for the efficient OER performance (Figure 4b). The N_2_‐NiFe‐PBA/NCF/CC‐60 delivers a lower overpotential of 270 mV at 50 mA cm^−2^ relative to N_2_‐NiFe‐PBA/NCF/CC‐30 (297 mV) and N_2_‐NiFe‐PBA/NCF/CC‐120 (295 mV), demonstrating that too many V_CN_ will destroy the structure of NiFe‐PBA and reduce its catalytic activity. Besides, N_2_‐NiFe‐PBA/NCF/CC‐60 exhibits more excellent OER performance compared to N_2_‐NiFe‐PBA/CC‐60, attributing to the abundant reactive site. The N_2_‐NiFe‐PBA/NCF/CC‐60 exhibits a lower overall overpotential (Δ*E*_all_ = *E*_OER/j_ *_=_*_50 mA cm_^−2^_–_ Δ*E*_ORR/j = 5 mA cm_^−2^) of 610 mV (Figure 4c) relative to NiFe‐PBA/NCF/CC (690 mV), N_2_‐NiFe‐PBA/NCF/CC‐30 (637 mV), and N_2_‐NiFe‐PBA/NCF/CC‐120 (635 mV), demonstrating the excellent bifunctional catalytic activity. Furthermore, the OER Tafel slopes of electrodes are calculated and follow in the order: N_2_‐NiFe‐PBA/NCF/CC‐60 (70 mV dec^−1^) < N_2_‐NiFe‐PBA/NCF/CC‐30 (75 mV dec^−1^) = Ir/C/CC (75 mV dec^−1^) < N_2_‐NiFe‐PBA/NCF/CC‐120 (76 mV dec^−1^) < N_2_‐NiFe‐PBA/CC‐60 (84 mV dec^−1^) < NiFe‐PBA/NCF/CC (122 mV dec^−1^), indicating the excellent OER catalytic reaction kinetics of N_2_‐NiFe‐PBA/NCF/CC‐60 (Figure 4d). Compared with NiFe‐PBA/NCF/CC, the N_2_‐NiFe‐PBA/NCF/CC‐X exhibit lower OER Tafel slopes, which shows the V_CN_ can effectively improve the OER catalytic reaction kinetics.

The ORR and OER durability of N_2_‐NiFe‐PBA/NCF/CC‐60 are evaluated via the chronoamperometric/chronopotentiometry method, respectively. The N_2_‐NiFe‐PBA/NCF/CC‐60 electrode exhibits a retention rate of the current density of 88% after 22 h ORR testing, higher than N_2_‐NiFe‐PBA/NCF/CC‐120 (82%) and NiFe‐PBA/NCF/CC (70%) (Figure 4e). The enhancement of potential of N_2_‐NiFe‐PBA/NCF/CC‐60 is observed shown in Figure 4f, which is much smaller than N_2_‐NiFe‐PBA/NCF/CC‐120 and NiFe‐PBA/NCF/CC. These results show the outstanding catalytic durability of N_2_‐NiFe‐PBA/NCF/CC‐60 toward ORR/OER. The Energy‐dispersive X‐ray spectroscopy (EDX) is conducted to analyze elements content after 15h OER testing. Figure S10 (Supporting Information) shows the Fe element is almost lost in NiFe‐PBA/NCF/CC, while N_2_‐NiFe‐PBA/NCF/CC‐60 only suffers a certain amount of iron loss. The SEM elemental mapping data of N_2_‐NiFe‐PBA/NCF/CC‐60 with NiFe‐PBA/NCF/CC after 15 h OER testing are obtained and shown in Figures S11 and S12 (Supporting Information), which agrees well with the EDX results. These results reveal that the V_CN_ can restrain the loss of Fe element during the OER process, thus improving the catalytic durability of N_2_‐NiFe‐PBA/NCF/CC‐60.

For demonstrating the superiority of flexible 3D free‐standing electrodes for electrocatalytic application, the p‐N_2_‐NiFe‐PBA/NCF/CC‐60 electrode is prepared by using Nafion as the binder and used for the control group (detailed scheme in the Experimental Section). As shown in Figure S13 (Supporting Information), the N_2_‐NiFe‐PBA/NCF/CC‐60 displays a higher current density of ORR at the same potential and lower potential of OER at the same current density relative to p‐N_2_‐NiFe‐PBA/NCF/CC‐60, proving the more efficient ORR/OER catalytic performance. As shown in Figure S14a–c (Supporting Information), the N_2_‐NiFe‐PBA/NCF/CC‐60 exhibits an electrochemical double‐layer capacitance (*C*_dl_) value of 243.6 mF cm^−2^, higher than p‐N_2_‐NiFe‐PBA/NCF/CC‐60 (186.2 mF cm^−2^), indicating the larger electrochemical active area of N_2_‐NiFe‐PBA/NCF/CC‐60 for catalyzing. This phenomenon is because binder (Nafion) is absorbed on the surface of the material, thus lowering catalytic reactive sites. The electrical conductivity of catalysts is investigated by electrochemical impedance spectroscopy (Figure S14d, Supporting Information). The N_2_‐NiFe‐PBA/NCF/CC‐60 displays a polarization resistance of 1.4 Ω, lower than p‐N_2_‐NiFe‐PBA/NCF/CC‐60 (3.6 Ω), revealing the excellent electrical conductivity. The introduced binder with poor electrical conductivity will increase the internal resistance, further impeding the charge/electron transfer process in the electrode. Therefore, the flexible binder‐free electrode with a 3D network structure not only provides efficient and sufficient ORR/OER reactive sites but also exhibits excellent electrical conductivity and avoids the side‐effect of the binder. Due to the above advantages, the N_2_‐NiFe‐PBA/NCF/CC‐60 electrode exhibits excellent bifunctional catalytic performance.

### 2.3 Liquid Zinc–Air Batteries and Flexible Zinc–Air Batteries Performance

**Figure 5:**
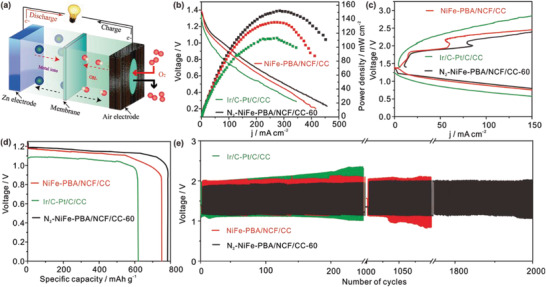
a) Schematic of liquid Zinc–air battery. b) Polarization curves and corresponding power density curves of liquid Zinc–air batteries. c) The charge/discharge polarization curves of batteries. The specific capacity curves d) and charge/discharge cycle curves e) at 10 mA cm^−2^.

As the schematic in **Figure** **5**a, a rechargeable liquid Zinc–air battery is constructed with N_2_‐NiFe‐PBA/NCF/CC‐60 as the air electrode to evaluate the practical application. It is concluded from Figure 5b that the Zinc–air batteries with N_2_‐NiFe‐PBA/NCF/CC‐60 deliver a power density of 155.0 mW cm^−2^, much higher than Ir/C‐Pt/C/CC (112.0 mW cm^−2^) and NiFe‐PBA/NCF/CC (135.0 mW cm^−2^) based batteries. Figure 5c shows the N_2_‐NiFe‐PBA/NCF/CC‐60 based battery exhibits a much lower overpotential of charge/discharge than NiFe‐PBA/NCF/CC and Ir/C‐Pt/C/CC based batteries at the same current density. Besides, the specific capacity of Zn–air batteries are calculated and follow the order (Figure 5d): N_2_‐NiFe‐PBA/NCF/CC‐60 (775 mAh g_Zn_^−1^) > NiFe‐PBA/NCF/CC (750 mAh g_Zn_^−1^) > Ir/C‐Pt/C/CC (620 mAh g_Zn_^−1^). This result shows the excellent battery performance of the liquid Zn–air battery, ascribed to the high‐efficiency OER/ORR performance of N_2_‐NiFe‐PBA/NCF/CC‐60. In addition, long‐term discharge/charge testing is conducted to further evaluate the battery durability (Figure 5e). The Zinc–air batteries with N_2_‐NiFe‐PBA/NCF/CC‐60 have been experienced for 2000 discharge/charge cycles and exhibit a slight increase of the voltage gap, while batteries with Ir/C‐Pt/C/CC and NiFe‐PBA/NCF/CC deliver a rapid increase of voltage gap and have successively lost battery performance, indicating the outstanding operational durability of N_2_‐NiFe‐PBA/NCF/CC‐60 air electrode. Figure S15 (Supporting Information) shows that N_2_‐NiFe‐PBA/NCF/CC‐60 based Zinc–air batteries manifest higher power density and more outstanding cyclic stability relative to p‐N_2_‐NiFe‐PBA/NCF/CC‐60 based batteries, demonstrating the flexible 3D free‐standing electrode benefits to improve battery performance. Such outstanding performance of Zn–air batteries with N_2_‐NiFe‐PBA/NCF/CC‐60 is superior to many reported Zinc–air batteries in previous literature (Table S1, Supporting Information).

**Figure 6:**
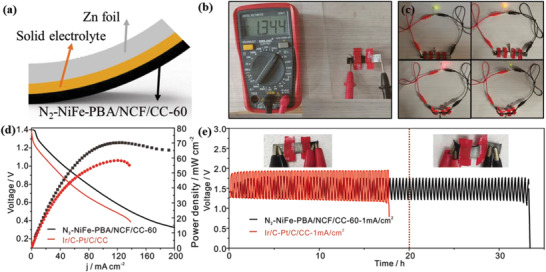
a) Schematic of flexible Zn–air battery. b) The open–circuit voltage of flexible Zn–air battery. c) The LEDs lit by two batteries in series. d) The polarization curves and power density curves of batteries. e) The long‐term discharge/charge cycle curves of batteries.

The rechargeable flexible Zn–air batteries are assembled by N_2_‐NiFe‐PBA/NCF/CC‐60, zinc foil anode and solid‐state electrolyte (**Figure** **6**a). A stable open–circuit voltage (1.34 V) of flexible Zn–air battery is observed in different bending states (Figure 6b; and Figure S16, Supporting Information). As shown in Figure 6c, a red/yellow/green light‐emitting diode (LED) is lit by two batteries and remains glowing when two batteries are deliberately bent. Two LEDs can be driven by three batteries in series (Figure S17, Supporting Information). Besides, the N_2_‐NiFe‐PBA/NCF/CC‐60 driven flexible batteries display a higher power density of 71 mW cm^−2^ compared to Ir/C‐Pt/C/CC (58 mW cm^−2^) (Figure 6d). The benign durability of the battery with N_2_‐NiFe‐PBA/NCF/CC‐60 in different bending states is verified via the galvanostatic charge/discharge cycles measurement (Figure 6e). The N_2_‐NiFe‐PBA/NCF/CC‐60 driven flexible battery delivers a low discharge/charge overpotential of 0.52 V at 1.0 mA cm^−2^ and exhibits a small voltage change after 33 h testing, while the battery with Ir/C‐Pt/C/CC exhibits a much higher voltage gap of 0.70 V and undergoes gradual degeneration within 17 h. It is known from Table S2 (Supporting Information) that N_2_‐NiFe‐PBA/NCF/CC‐60 based flexible battery exhibits more excellent performance than many previous batteries, confirming the excellent performance of N_2_‐NiFe‐PBA/NCF/CC‐60 based flexible Zn–air battery.

### 2.4 Self‐Driven Water‐Splitting Unit Performance

**Figure 7:**
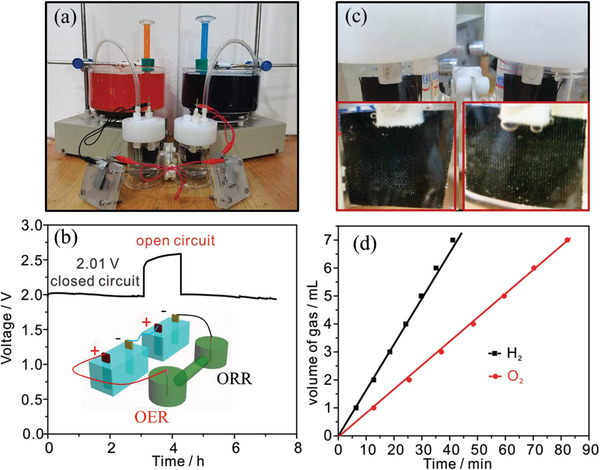
a) The water‐splitting device driven by liquid Zinc–air batteries. b) The images and the enlarged images (inset) of two electrodes during water splitting. The voltage‐time curves c) and the volume of collected gas‐time curves d).

As shown in **Figure** **7**a, a water‐splitting unit is constructed by Pt/C/CC cathode, N_2_‐NiFe‐PBA/NCF/CC‐60 anode, and 1 m KOH electrolyte, which is driven by two N_2_‐NiFe‐PBA/NCF/CC‐60 based liquid batteries. An output voltage of about 2.01 V of two Zn–air batteries is observed and applied to drive electrolysis of water at the closed‐circuit state and exhibits a slight decrease after about 6 h operation, indicating the excellent durability of the water‐splitting unit (Figure 7b). The O_2_/H_2_ bubbles are observed on the anode/cathode and collected by the drainage collection method (Figure 7c). As shown in Figure 7d, the slopes of volume‐time curves for O_2_ and H_2_ are calculated to be 1.18 and 2.35 µL s^−1^, respectively. In all, such excellent performance manifests the promising practicability of water‐splitting units driven by N_2_‐NiFe‐PBA/NCF/CC‐60 based liquid batteries.

## 3 Conclusions

In summary, a flexible free‐standing air‐electrode is synthesized by the 3D spatial combination of CN vacancy‐mediated NiFe‐PBA with N‐doped carbon fibers rooted on CC. The in situ formed V_CN_ in NiFe‐PBA by N_2_‐plasma activation is demonstrated by multiple characterization techniques. The V_CN_ can tune the local coordination environment and electronic structure of the Ni‐Fe active sites in NiFe‐PBA, which greatly improves the OER catalytic intrinsic activity of N_2_‐NiFe‐PBA/NCF/CC‐60 and restrains the loss of Fe element during the OER process. The 3D spatial combination of CN vacancy‐mediated NiFe‐PBA with N‐doped carbon fibers not only guarantees a large specific surface and excellent electrical conductivity but also provides efficient, stable and sufficient ORR/OER active sites; and avoids the side‐effect of the binder. Therefore, the N_2_‐NiFe‐PBA/NCF/CC‐60 electrode delivers efficient bifunctional catalytic activity and excellent durability. Besides, the N_2_‐NiFe‐PBA/NCF/CC‐60 based liquid Zinc–air battery exhibits outstanding charge/discharge stability for 2000 cycles and a high powers density of 155.0 mW cm^−2^. The corresponding flexible Zinc–air battery exhibits excellent mechanical properties and battery performance. In addition, a water‐splitting device can be driven stably for 6 h by two N_2_‐NiFe‐PBA/NCF/CC‐60 based liquid Zinc–air batteries. Therefore, the present study provides an effective strategy for flexible free‐standing electrodes by manipulating defect chemistry and designing microstructure, which promotes the performance of flexible Zinc–air batteries.

## Conflict of Interest

The authors declare no conflict of interest.

## Supporting information

Supporting Information

## Data Availability

Research data are not shared.
