# Supplementary material for: 3D Spatial Combination of CN Vacancy‐Mediated NiFe‐PBA with N‐Doped Carbon Nanofibers Network Toward Free‐Standing Bifunctional Electrode for Zn–Air Batteries
Source: Adv Sci (Weinh). 2022 Feb 22;9(11):2105925. doi: 10.1002/advs.202105925 (PMC9008428; doi:10.1002/advs.202105925)
Supplement: Supplementary file 1 — Supporting Information [file ADVS-9-2105925-s001.pdf]

ADVANCED SCIENCE
Open Access

Supporting Information

for *Adv. Sci.*, DOI 10.1002/advs.202105925

3D Spatial Combination of CN Vacancy-Mediated NiFe-PBA with N-Doped Carbon Nanofibers Network Toward Free-Standing Bifunctional Electrode for Zn–Air Batteries

Chenglong Lai, Haomiao Li, Yi Sheng, Min Zhou, Wei Wang, Mingxing Gong, Kangli Wang and Kai Jiang**

3D spatial combination of CN vacancy-mediated NiFe-PBA with N-doped carbon nanofibers network toward free-standing bifunctional electrode for Zn-air batteries driven water splitting

Chenglong Lai[a, b‡], Haomiao Li[a, c, ‡], Yi Sheng [a, b], Min Zhou[a, c], Wei Wang[b, c], Mingxing Gong[d], Kangli Wang [a, c, *], Kai Jiang[a, c, *]

[a] *State Key Laboratory of Advanced Electromagnetic Engineering and Technology, School of Electrical and Electronic Engineering, Huazhong University of Science and Technology, Wuhan 430074, P. R. China.*

[b] *School of Materials Science and Engineering, Huazhong University of Science and Technology, Wuhan, Hubei 430074, China*

[c] *Engineering Research Center of Power Safety and Efficiency, Ministry of Education，Wuhan, Hubei 430074, China*

[d]*Engineering Research Center of Nano-Geomaterials of Ministry of Education, Faculty of Materials Science and Chemistry, China University of Geosciences, Wuhan 430078.*

‡ *These authors contributed equally to this work*

* *Corresponding author*

1. Experimental Section

1.1. Catalysts preparation

Synthesis of PNF/CC: The hydrophilic carbon cloth was obtained by soaking in concentrated nitric acid for 6h. The hydrophilic carbon cloth, saturated calomel electrode and carbon rod were selected as working electrode, reference electrode and counter electrode, respectively. The uniform electrolyte was formed by adding 0.532 g of $LiClO_4$, 1.06 g of Na_2CO_3 and 1.0 mL of pyrrole into 50 mL of H_2O. The PNF/CC

was prepared by constant voltage electrodeposition at the oxidation current density of 10 mA/cm^2 last for 30 min.

Synthesis of NCF/CC: The NCF/CC was synthesized by pyrolyzing PNT/CC at 800 °C for 2 h under Ar atmosphere.

Synthesis of NiFe-PBA/NCF/CC: Firstly, the solution A was prepared by adding 0.3971 g of $Na_3C_6H_5O_7·2H_2O$ and 0.2139 g of $Ni(NO_3)_2•6H_2O$ into 30 mL of H_2O. The 0.1976 g of $K_3[Fe(CN)_6]$ was dispersed in 30 mL of H_2O to form solution B. Secondly, the solutions A and B were mixed together, the *NiFe-PBA/NCF/CC* was synthesized by soaking NCF/CC in mixed solution for 10 h. Besides, the NiFe-PBA/CC was prepared under the same procedure by replacing PNF/CC with CC.

Synthesis of N_2-NiFe-PBA/NCF/CC-X: When the plasma power is 100 W and the pressure is 3 mTorr, the NiFe-PBA/NCF/CC was treated by N_2 plasma at various irradiation times of 30 min, 60 min, and 120 min to obtain N_2-NiFe-PBA/NCF/CC-30, N_2-NiFe-PBA/NCF/CC-60 and N_2-NiFe-PBA/NCF/CC-120, respectively. The NiFe-PBA/CC was treated by N_2 plasma for 60 min to form the N_2-NiFe-PBA/CC-60.

Synthesis of p-N_2-NiFe-PBA/NCF/CC-60: A piece of NiFe/NCNF/CC was shredded, ground into fine powder, the ink was formed by mixing powder and 0.1%wt Nafion isopropyl alcohol solution. The p-N_2-NiFe-PBA/NCF/CC-60 was obtained by dropping ink evenly onto the carbon cloth with the same size.

Synthesis of *Pt/C/CC and Ir/C/CC*: The ink was formed by adding 0.128 mg of 20 wt% Pt/C into 0.05 mL of 0.1%wt Nafion solution. The Pt/C/CP was prepared by dropping the ink evenly onto the carbon cloth (1*1 cm^2). The loading of Pt/C is 0.128 mg/cm^2. The Ir/C/CP was prepared by the same procedure with a loading of 0.42 mg/cm^2.

Synthesis of Pt/C-Ir/C/CC: A ink was formed by adding 0.5 mg of 20 wt% Ir/C and 0.5 mg of 20 wt% Pt/C into 0.2 mL of 0.1%wt Nafion solution. The Pt/C-Ir/C/CC was prepared by dropping ink evenly onto the carbon cloth (1cm * 1cm).

1.2．Physical characterization

The scanning electron microscopy (SEM, Sirion200) was selected to characterized the

morphologies of catalysts. The X'Pert PRO diffractometer was conducted to obtain Powder X-ray diffraction (XRD) patterns. The XPS data were obtained by a ThermoFischer, ESCALAB Xi+ Instrument.

1.3. Electrochemical measurement

The carbon rod, synthesized catalysts (air-electrode) and reverse hydrogen electrode are selected as the counter, working and reference electrodes, respectively. The electrochemical measurement is conducted in O_2/N_2 saturated 1.0 KOH electrolyte. The rate of 50 mV s^{-1} and 5 mV s^{-1} were selected to operate CV testing and LSV testing, respectively.

1.4. Fabrication of Liquid Zn-air batteries

The liquid Zinc-air battery is assembled by polished Zn plate anode, N_2-NiFe-PBA/NCF/CC-60 air-electrode and electrolyte. The electrolyte is 6.0 M KOH + 0.2 M $Zn(CH_3COO)_2$ mixed solution. The LAND-CT2001A tester was conducted to evaluate the performance of liquid Zinc-air batteries.

1.5. Fabrication of flexible Zn-air batteries

The flexible Zn-air battery consisted of zinc foil anode, solid-state electrolyte and N_2-NiFe-PBA/NCF/CC-60 air-cathode is constructed and shown in Figure 5a. The solid-state electrolyte is synthesized by following procedure. First, synthesized 8 mL of 11.25 M KOH and 0.25 M ZnO mixed solution, added 0.8 g of acrylic acid and 0.12 g of N,N'-methylene-bisacrylamide into it, and sonication for 10 min. Then, the solution solidified into an electrolyte after adding 120 µL of 0.30 M $K_2S_2O_4$.

1.6. Self-made water-splitting unit

The self-made water-splitting device consisted of Pt/C/CC cathode and N_2-NiFe-PBA/NCF/CC-60 anode was assembled and driven by two liquid Zn-air batteries. The drainage collection method was selected to collect the generated O_2 and H_2.

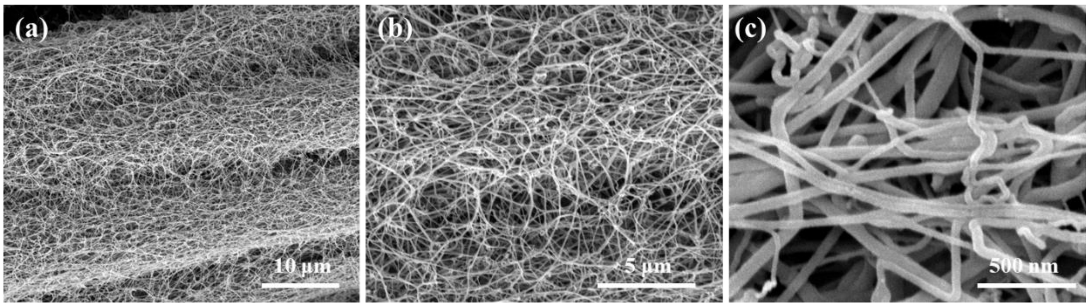

Figure S1. The SEM images of NCF/CC.

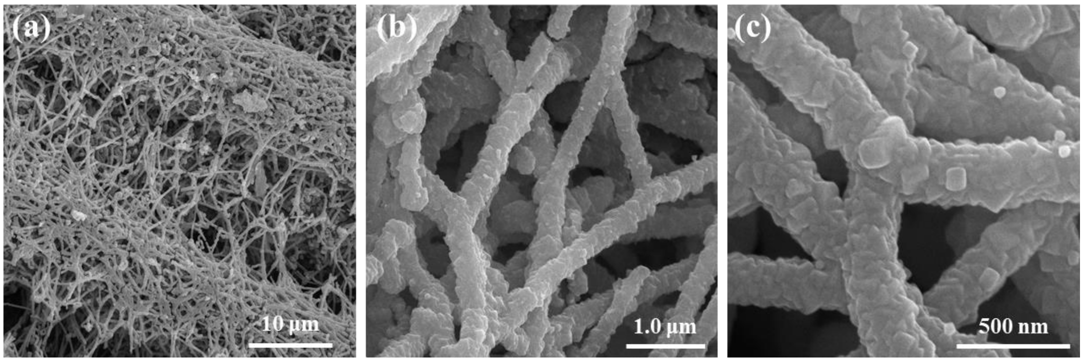

Figure S2. The SEM images of NiFe-PBA/NCF/CC.

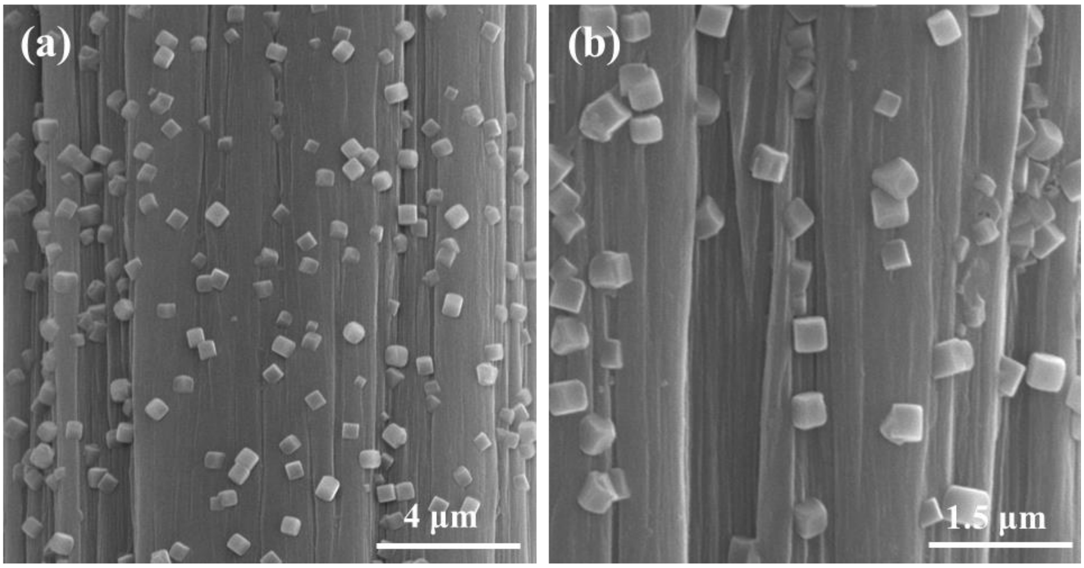

Figure S3. The SEM images of NiFe-PBA/CC.

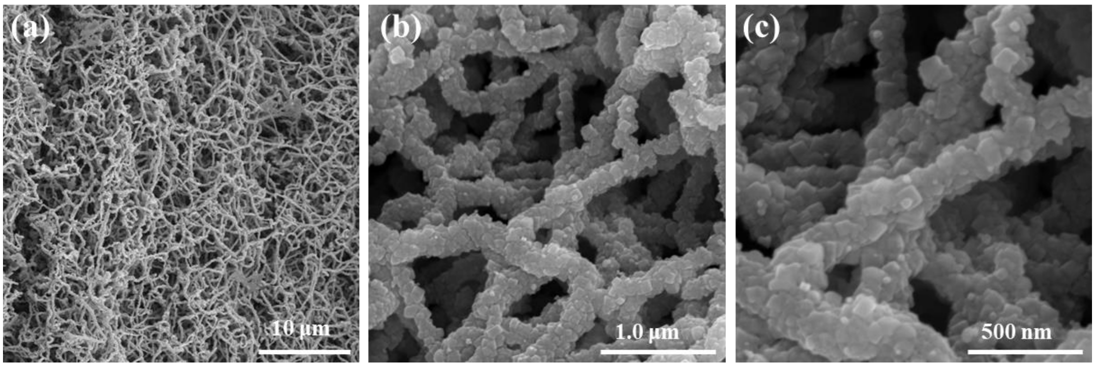

Figure S4. The SEM images of N_2-NiFe-PBA/NCF/CC-30.

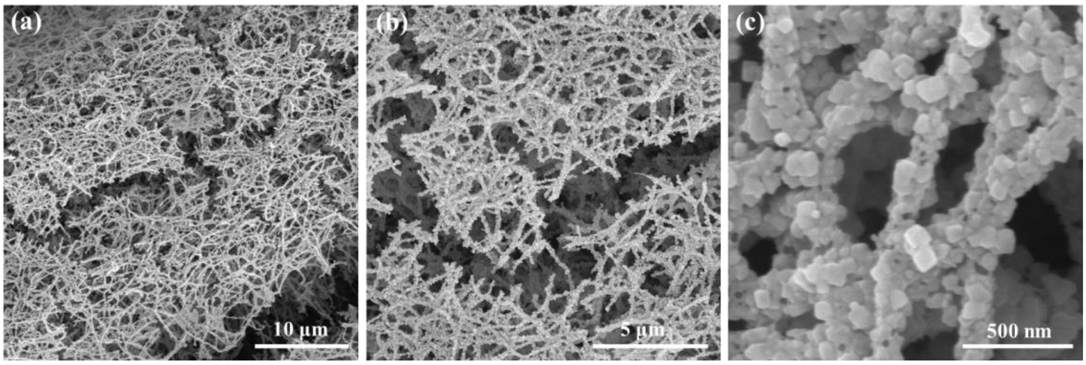

Figure S5. The SEM images of N_2-NiFe-PBA/NCF/CC-120.

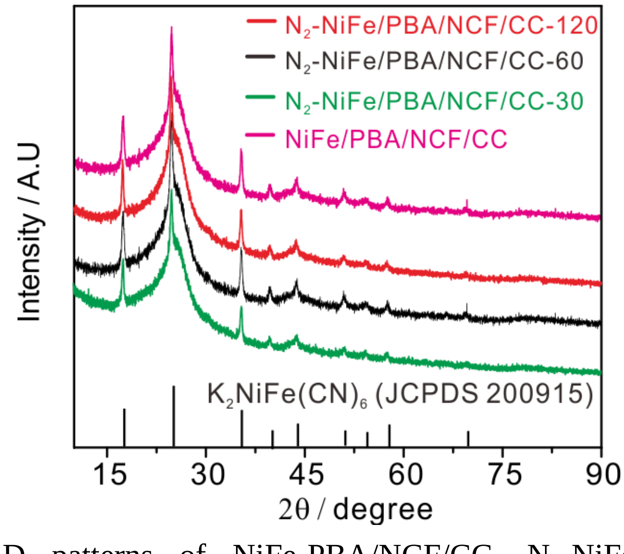

Figure S6. XRD patterns of NiFe-PBA/NCF/CC, N_2-NiFe-PBA/NCF/CC-30, N_2-NiFe-PBA/NCF/CC-60 and N_2-NiFe-PBA/NCF/CC-120.

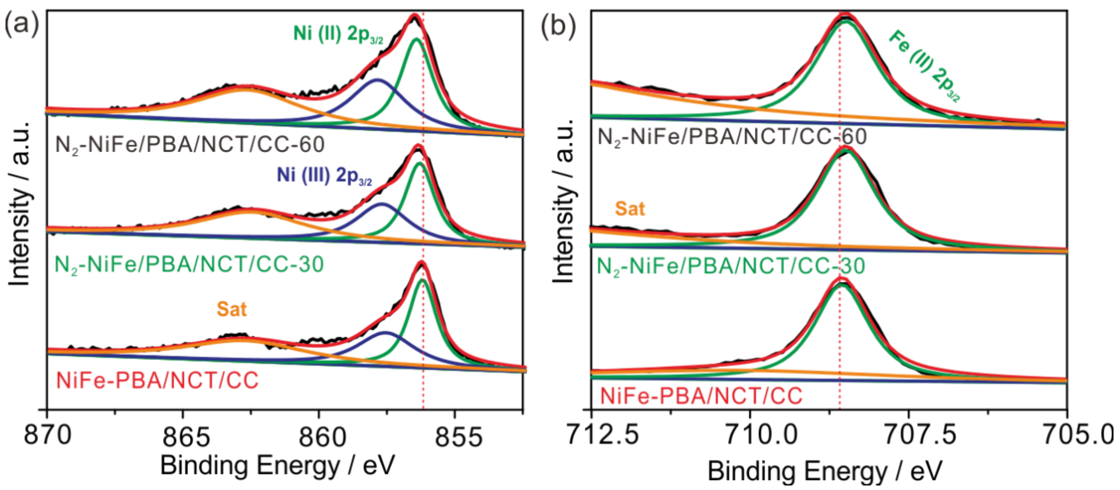

Figure S7. The fine Ni spectra (e) and Fe spectra (f) of NiFe-PBA/NCF/CC and N_2-NiFe-PBA/NCF/CC-X.

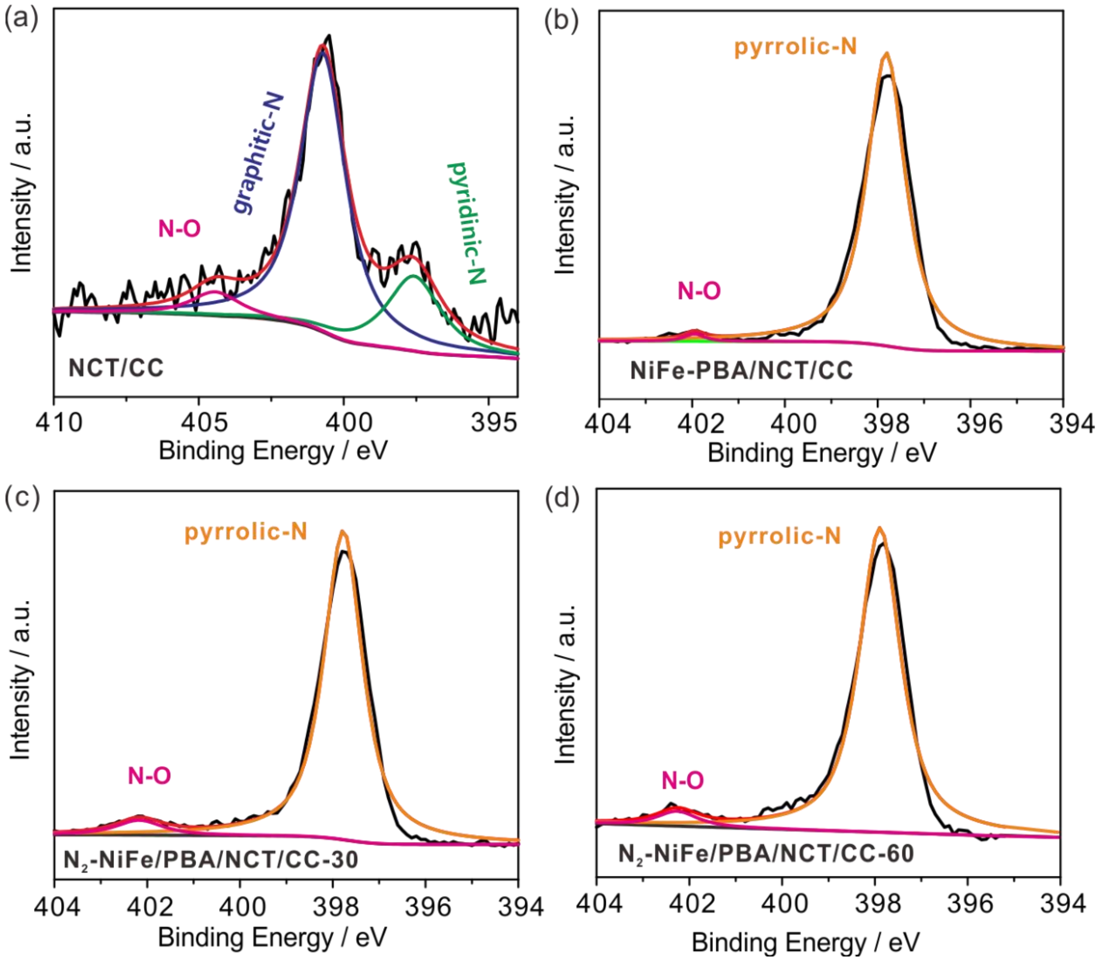

Figure S8. The fine N spectra of NCT/CC (a), NiFe-PBA/NCF/CC(b), N_2-NiFe-PBA/NCF/CC-30 (c) and N_2-NiFe-PBA/NCF/CC-60 (d).

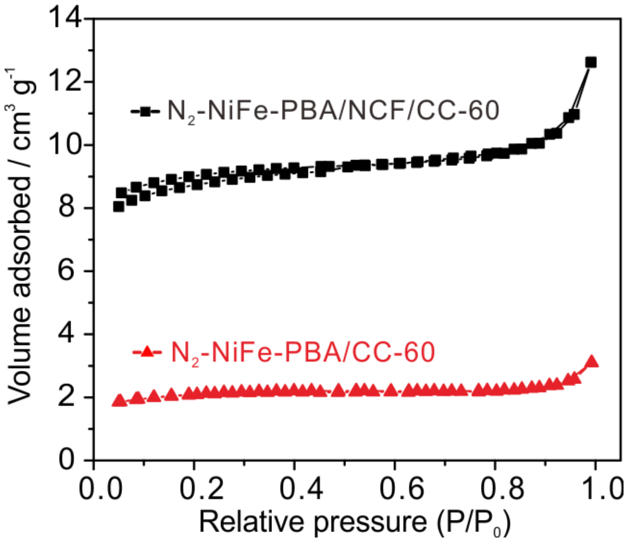

Figure S9. N_2 adsorption-desorption isotherms for N_2-NiFe-PBA/NCF/CC-60 and N_2-NiFe-PBA/CC-60.

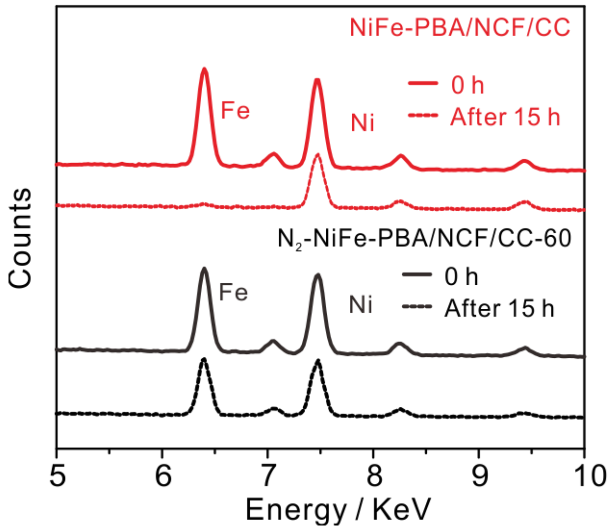

Figure S10. EDX spectra of N_2-NiFe-PBA/NCF/CC-60 and NiFe-PBA/NCF/CC catalysts that cycled for 15 h OER testing.

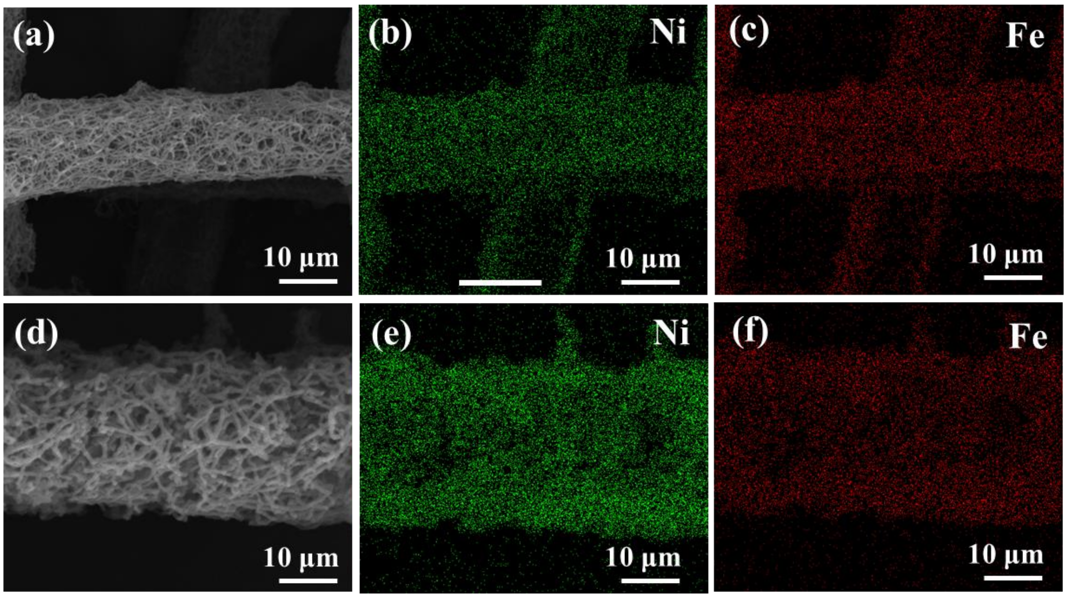

Figure S11. EDX elemental mappings of N_2-NiFe-PBA/NCF/CC-60 and N_2-NiFe-PBA/NCF/CC-60 after 15 h OER testing.

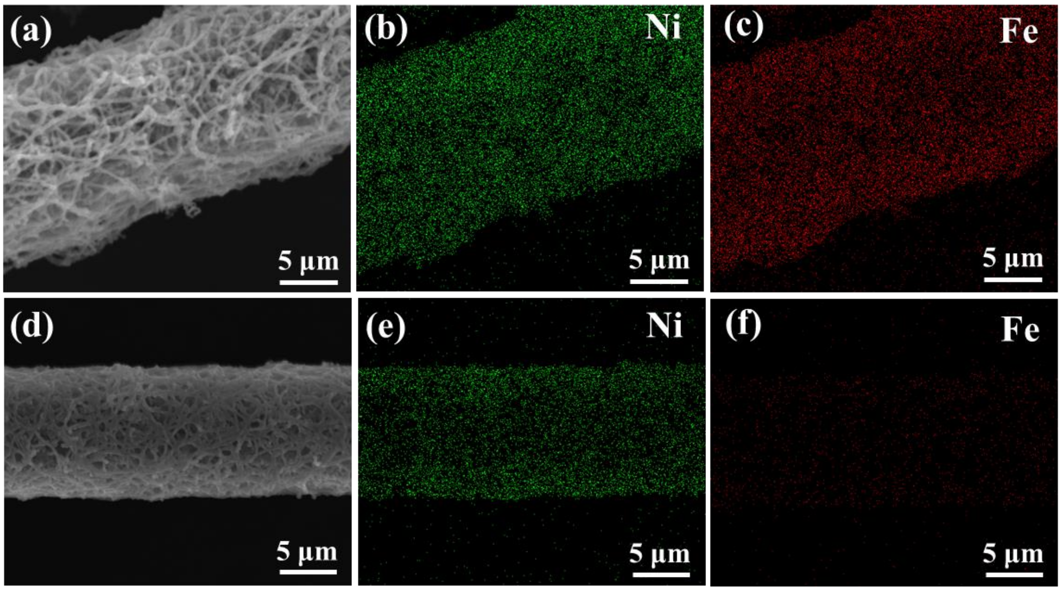

Figure S12. EDX elemental mappings of NiFe-PBA/NCF/CC and NiFe-PBA/NCF/CC after 15 h OER testing.

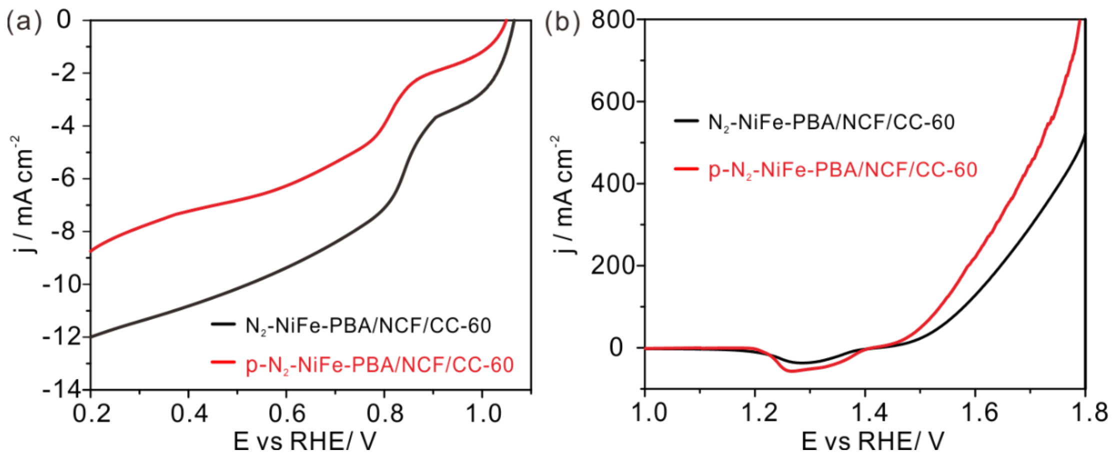

Figure S13. The ORR (a) and OER (b) polarization curves of N_2-NiFe-PBA/NCF/CC-60 and p-N_2-NiFe-PBA/NCF/CC-60.

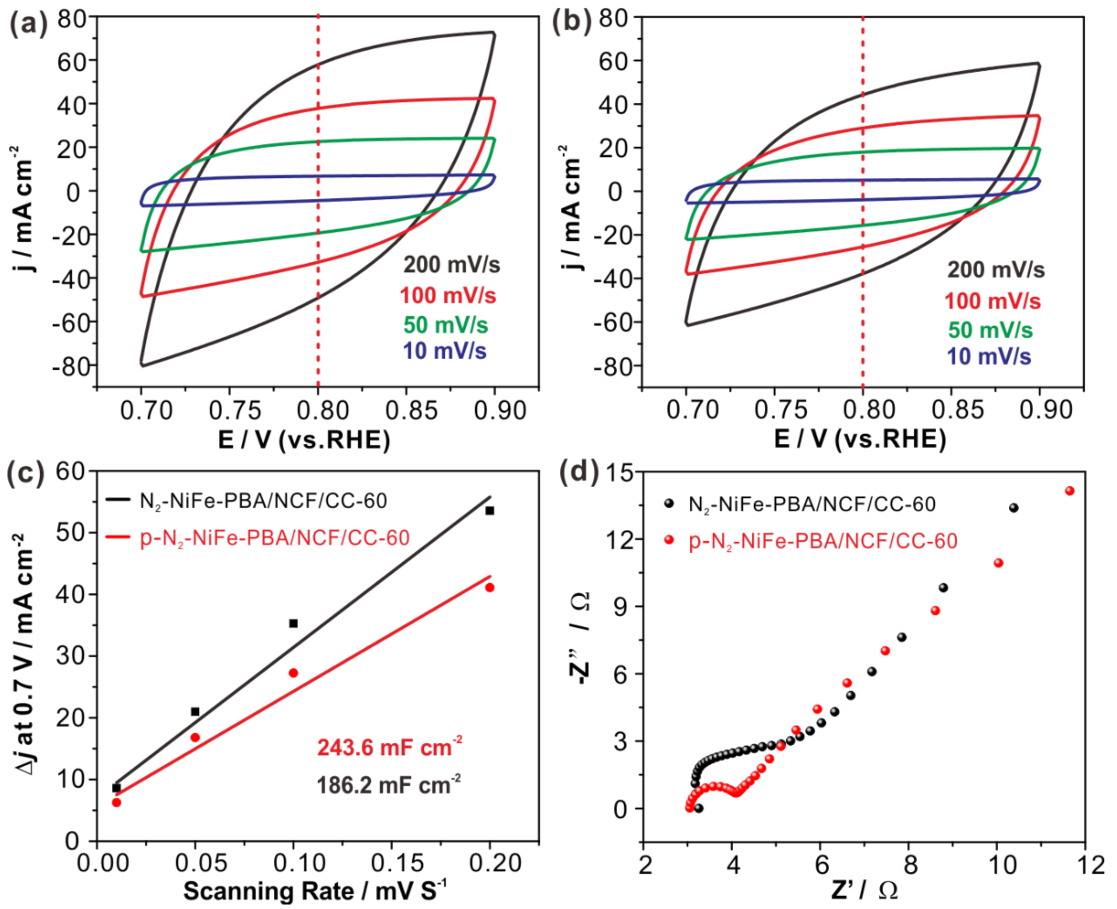

Figure S14. The CV curves between 0.7 and 0.9 V at different scan rates of N_2-NiFe-PBA/NCF/CC-60 (a) and p-N_2-NiFe-PBA/NCF/CC-60 (b). (c) The half of current density variation ($\Delta J = (/J_a/ + /J_c/)/2$) at 0.8V plotted against scan rate derived from Figure S14(a-b). (d) The Nyquist plots of N_2-NiFe-PBA/NCF/CC-60 and p-N_2-NiFe-PBA/NCF/CC-60.

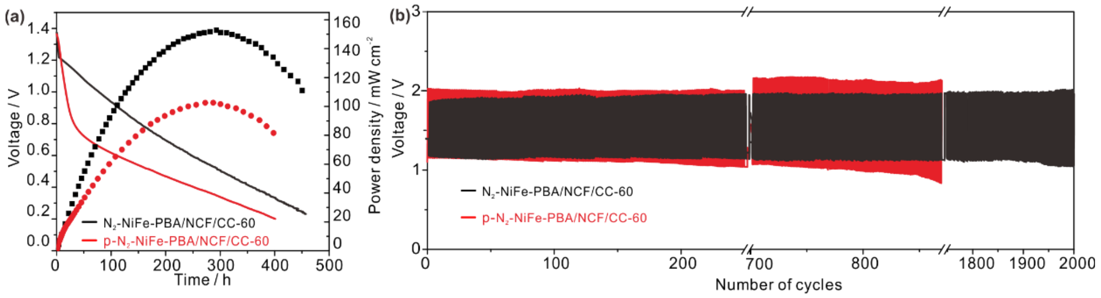

Figure S15. (a) The polarization curves and corresponding power density curves of N_2-NiFe-PBA/NCF/CC-60 and p-N_2-NiFe-PBA/NCF/CC-60. (b) The galvanostatic charge/discharge cycle curves.

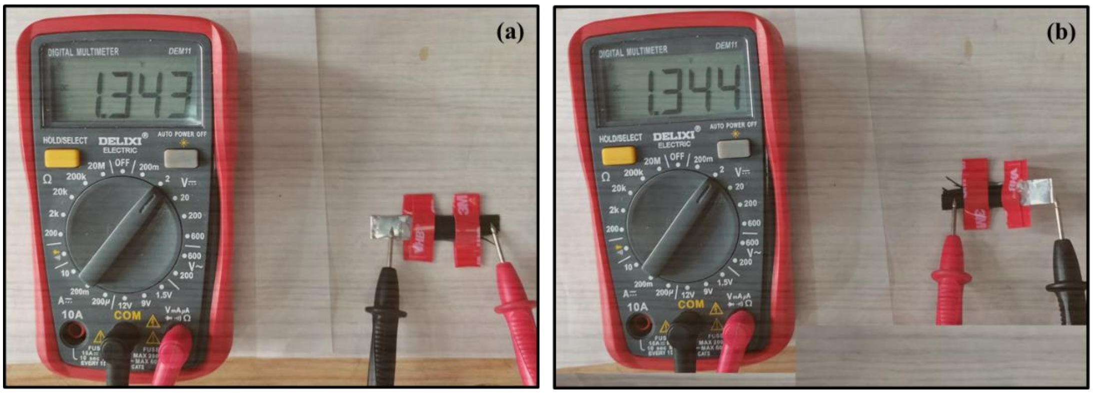

Figure S16. (a-b) The open-circuit voltage of battery in different bending states.

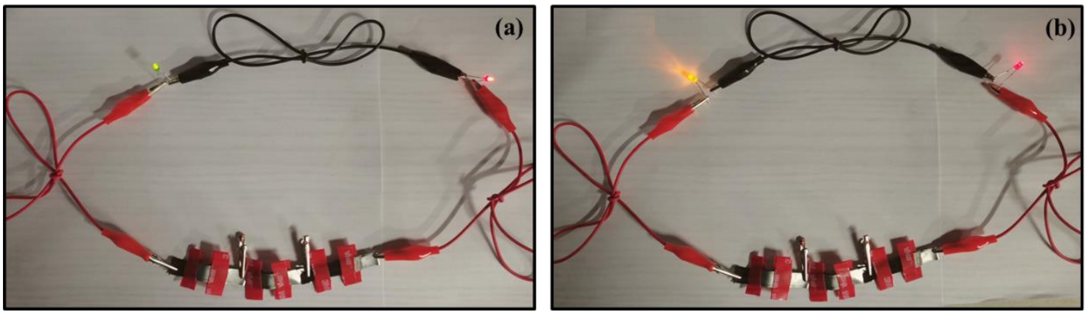

Figure S17. Two LEDs are lit by three batteries in series.

Table S1. Comparison of key performance parameters for rechargeable Zn-air batteries extracted from literatures.

Catalysts	Peak power density	Voltage gap	Electrolyte	Refs
N_2-NiFe-PBA/NCF/CC-60	155.5 mW cm^{-2}	0.75 V@ 10 mA cm^{-2}	6 M KOH + 0.2M zinc acetate	This work
nNiFe LDH/3D MPC	97 mW cm^{-2}	0.82 V @ 10 mA cm^{-2}	6 M KOH + 0.2M zinc acetate	1
$CoO_{0.87}S_{0.13}$/GN	100 mW cm^{-2}	0.76 V@ 20 mA cm^{-2}	6 M KOH + 0.2M zinc acetate	2
$Co_3FeS_{1.5}(OH)_6$	113.1 mW cm^{-2}	0.84 V @ 2 mA cm^{-2}	6 M KOH + 0.2M $ZnCl_2$	3
C-MOF-C_2-900	105 mW cm^{-2}	0.53 V @ 2 mA cm^{-2}	6 M KOH + 0.2M zinc acetate	4
NiFe@NBCNT	132 mW cm^{-2}	0.8 V @ 10 mA cm^{-2}	6 M KOH + 0.2M $ZnCl_2$	5
$Fe_{0.5}Co_{0.5}O_x$/NrGO	86 mW cm^{-2}	0.79 V@ 10 mA cm^{-2}	6 M KOH + 0.2M $ZnCl_2$	6
NC-Co_3O_4-90	---	0.8 V @ 5 mA cm^{-2}	6 M KOH + 0.2M zinc acetate	7

Table S2. Comparison of key performance parameters for flexible Zn-air batteries extracted from literatures.

Catalysts	Electrode configuration	Peak power density	Cycling ablity	Solid state electrolyte	Refs
N_2-NiFe-PBA/NCF/CC-60	Sandwich	71.0 mW cm^{-2}	99 cycles (33 h) @1.0 mA cm^{-2}	PAM/KOH	This work
CoFe/N-GCT	Sandwich	---	95 cycles (16 h) @2.0mA cm^{-2}	PVA/KOH	8
FeNx-PNC	Sandwich		220 cycles (40 h)	PAM/KOH	9
Co_3O_4@Co/NCNT	Sandwich		16 cycles (1 h) @2.0mA cm^{-2}	PVA/KOH	10
N-GQDs/$NiCo_2S_4$/CC	Sandwich	26.2 mW cm^{-2}	36 cycles (12 h)	PVA/KOH	11

Co-SAs@NC	Sandwich		35 cycles (700 min) @2.0mA cm^{-2}	PVA/KOH	12
Co_3O_4/N-rGO	Cable-type		75 cycles (25 h) @2.0mA cm^{-2}	PVA/KOH	13
Co_4N/CNW/CC	Cable-type		36 cycles (12 h) @1.0mA cm^{-2}	PVA/KOH	14
$FeCo_4N$@N-C	Sandwich	72 mW cm^{-2}	45 cycles @4.0mA cm^{-2}	PAM/KOH	15

[1] W. Wang, Y. Liu, J. Li, J. Luo, L. Fu, S. Chen. *J. Mater. Chem. A*, **2018**, *6*, 14299-14306.

[2] J. Fu, F. M. Hassan, C. Zhong, J. Lu, H. Liu, A. Yu, Z. Chen, *Adv. Mater.* **2017**, *29* 1702526.

[3] H. F. Wang, C. Tang, B. Wang, B. Q. Li, Q. Zhang, *Adv. Mater.* **2017**, *29*, 1702327.

[4] M. Zhang, Q. Dai, H. Zheng, M. Chen, L. Dai, *Adv. Mater.* **2018**, *30*, 1705431.

[5] D. Bin, B. Yang, C. Li, Y. Liu, X. Zhang, Y. Wang, Y. Xia, *ACS Appl. Mater. Inter.* **2018**, *10*, 26178-26187.

[6] L. Wei, H. E. Karahan, S. Zhai, H. Liu, X. Chen, Z. Zhou, Y. Lei, Z. Liu, Y. Chen, *Adv. Mater.* **2017,** *29*, 1701410.

[7] C. Guan, A. Sumboja, H. Wu, W. Ren, X. Liu, H. Zhang, Z. Liu, C. Cheng, S. J. Pennycook, J. Wang, *Adv. Mater***.** **2017**, *29* 1704117.

[8] X. Liu, L. Wang, P. Yu, C. Tian, F. Sun, J. Ma, W. Li, H. Fu, *Angew. Chem., Int. Ed.* **2018**, *57*, 16166–16170.

[9] L. Ma, S. Chen, Z. Pei, Y. Huang, G. Liang, F. Mo, Q. Yang, J. Su, Y. Gao, J. A.

Zapien, C. Zhi, *ACS Nano*, **2018**, **12**, 1949–1958.

[10] T. Singh, C. Das, N. Bothra, N. Sikdar, S. Das, S. K. Pati, T. K. Maji, *Inorg. Chem.* **2020** *59*, 3160-3170.

[11] W. Liu, B. Ren, Z. Chen, *Small* **2019**, *15*, 1903610.

[12] X. Han, X. Ling, Y. Wang, T. Ma, C. Zhong, W. Hu, Y. Deng, *Angew. Chem., Int. Ed.*, **2019**, *58*, 5359–5364.

[13] Y. Li, C. Zhong, J. Liu, X. Zeng, S. Qu, X. Han, Y. Deng, W. Hu, J. Lu, *Adv. Mater.*, **2018**, *30*, 1–9.

[14] F. Meng, H. Zhong, D. Bao, *J. Am. Chem. Soc*. **2016**, *138*, 10226-10231.

[15] Q. Xu, H. Jiang, C. Li, *Appl. Catal. B-Environ.* **2019**, *256, 117893.*
